# Metabolic Reprogramming and Cell Interaction in Atherosclerosis: From Molecular Mechanisms to Therapeutic Strategies

**DOI:** 10.3390/jcdd12100384

**Published:** 2025-09-28

**Authors:** Yu-Xin Liu, Feng-Ming Guo, Wen-Jun Qiu, Yi-Peng Gao, Xue-Yu Han, Bo Shen

**Affiliations:** 1 Department of Cardiology, Renmin Hospital of Wuhan University, Wuhan 430060, China; 2024283020048@whu.edu.cn (Y.-X.L.); fengming_guo@whu.edu.cn (F.-M.G.); 2023283020077@whu.edu.cn (W.-J.Q.); 2020305232001@whu.edu.cn (Y.-P.G.); 2025183020058@whu.edu.cn (X.-Y.H.); 2 Hubei Key Laboratory of Metabolic and Chronic Diseases, Wuhan 430060, China; 3 State Key Laboratory of Neurology and Oncology Drug Development, Nanjing 210000, China

**Keywords:** atherosclerosis, macrophage, endothelial cell, vascular smooth muscle cell, glucose and lipid metabolism, pharmacologic treatment

## Abstract

Atherosclerosis is a complex systemic inflammatory metabolic disease, which originates from endothelial dysfunction and progresses through plaque formation involving vascular smooth muscle cells (VSMCs) and macrophage uptake of modified low-density lipoprotein (LDL). These processes lead to vascular stenosis, plaque rupture, and potentially sudden death. Metabolic dysregulation and cellular remodeling are fundamental to the pathogenesis of atherosclerosis. In this review, we summarize recent advances in the metabolic reprogramming of major cell types (including endothelial cells, VSMCs, and macrophages) during atherosclerosis progression. Furthermore, we discuss the crosstalk among these cells mediated by such metabolic alterations. Finally, we highlight the implications of metabolic reprogramming for targeted therapeutic strategies, offering insights for precision intervention in aortic atherosclerosis.

## 1. Atherosclerosis

Atherosclerosis is currently regarded as a chronic inflammatory disease of the arterial wall. Risk factors of the disease include smoking, high blood pressure, dyslipidemia, and insulin resistance/hyperglycemia [1,2,3]. In addition, a number of novel metabolic causes of atherosclerosis have received widespread attention, including metabolic inflammation, hyperhomocysteinemia, and trimethylamine N-oxide (TMAO) [4,5,6]. Atherosclerosis typically has no symptoms in its early stages, but late thrombotic events can cause disability or even death. Ischemic heart disease, ischemic stroke, and peripheral arterial disease are the three most common clinical manifestations of atherosclerosis, and their incidence is typically increasing worldwide [7]. Overall, the global public economic and health burden of atherosclerosis keeps rising.

During the course of atherosclerotic lesions, lipids, extracellular matrix, and cellular debris accumulate beneath the endothelium within the tunica intima of the artery. This process is mediated by a variety of cells, primarily involving vascular endothelial cells (ECs), macrophages, and VSMCs [8]. When ECs are damaged, the permeability of ECs is increased, and LDL enters the tunica intima and is oxidized to oxidized low-density lipoprotein (ox-LDL). Ox-LDL triggers endothelial activation, prompting them to express adhesion molecules that recruit circulating monocytes. These monocytes adhere to the endothelium, migrate into the tunica intima, and differentiate into macrophages. The macrophages then uptake ox-LDL via scavenger receptors, transforming into lipid-laden foam cells—the hallmark of early atherosclerotic lesions. The subsequent death of these foam cells perpetuates inflammation and releases lipids. VSMCs play a dual role in atherosclerosis. They can stabilize plaques by secreting collagen and forming a fibrous cap, but they can also contribute to disease progression by forming foam cells and participating in the expansion of the necrotic core. The phenotypic switching of VSMCs is initiated almost concurrently with monocyte recruitment, following the endothelial expression of adhesion molecules. In the advanced stages, inflammatory factors released from dying foam cells potently stimulate VSMCs activation and migration, creating a vicious cycle that exacerbates atherosclerotic lesions.

Notably, the activation, proliferation, and function of these key cells within the atherosclerotic plaque are intrinsically linked to profound alterations in their glucose and lipid metabolism. This metabolic reprogramming not only supports the immense bioenergetic and biosynthetic demands of these cells, but also directly influences their inflammatory response and survival, thereby playing a crucial role in the progression and destabilization of plaques. Therefore, targeting cellular metabolism has emerged as a promising new strategy for the treatment of atherosclerosis. In this review, we will explore potential pharmacological interventions targeting the metabolic alterations in the major cells of atherosclerotic lesions. Our primary focus will be on ECs, VSMCs, and macrophages, with particular emphasis on the critical role of intercellular communication in driving disease progression.

## 2. Glucose and Lipid Metabolism of ECs in Atherosclerosis

**Figure 1:**
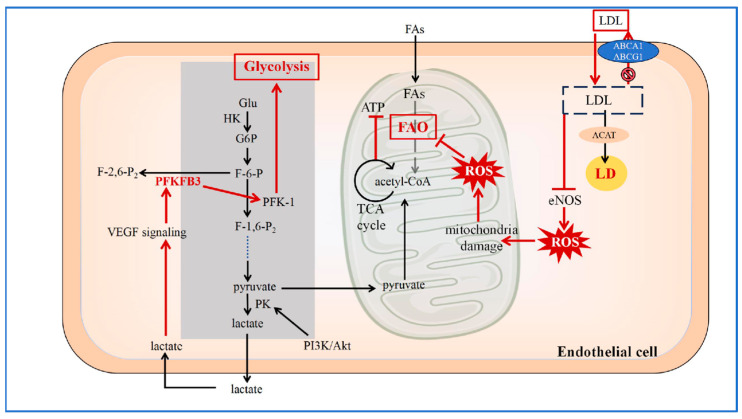
Reprogramming of Glucose and Lipid Metabolism in ECs.

The dysfunction of ECs represents the initiating event in the development of atherosclerotic lesions. ECs and the subendothelial layer make up the majority of the tunica intima. A hallmark of EC dysfunction is impaired endothelium-dependent vasodilation, which is characterized by reduced nitric oxide (NO) bioavailability, elevated reactive oxygen species (ROS), and increased expression of inflammatory mediators—a process that has been extensively studied [9]. Furthermore, angiogenesis is intimately linked to EC metabolism. Key metabolic pathways such as glycolysis and fatty acid oxidation provide not only energy but also biosynthetic intermediates essential for vascular sprouting and proliferation. For instance, glycolytic flux regulates endothelial nitric oxide synthase (eNOS) activity and redox balance, thereby influencing both vascular tone and inflammatory responses [10]. Given their central role in early atherogenesis and metabolic regulation, ECs serve as a primary focus in this review. We will examine both their physiological metabolic programs and the reprogramming that occurs under atherogenic conditions. The overall changes in glucose and lipid metabolism of endothelial cells are roughly shown in Figure 1.

In the atherosclerotic lesion area, endothelial cells undergo metabolic reprogramming in glycolipid metabolism. After LDL uptake, functional impairment of the cholesterol efflux transporters ABCA1 and ABCG1 leads to intracellular LDL accumulation. A portion of the accumulated LDL is esterified by ACAT and stored in LDs, a process that can promote endoplasmic reticulum stress and cellular injury. Concurrently, accumulated LDL inhibits eNOS activity, resulting in increased ROS levels. The elevated ROS contribute to mitochondrial dysfunction, impairing processes such as FAO and ATP production. In response, the glycolytic flux in endothelial cells is enhanced. The glycolytic product lactate can potentiate VEGF signaling and upregulate PFKFB3. Upregulated PFKFB3 enhances the activity of PFK-1, thereby further increasing glycolytic flux and creating a feed-forward loop. (LDL: Low-Density Lipoprotein; ACAT: acyl-CoA cholesterol acyltransferase; LD: lipid droplet; ROS: reactive oxygen species; eNOS: endothelial nitric oxide synthase; ABCA1: ATP-binding cassette transporters A1; ABCG1: ATP-binding cassette transporters G1; Glu: glucose; HK: hexokinase; G6P: glucose-6-phosphate; F-6-P: fructose 6-phosphate; PFK-1: phosphofructokinase-1; PFKFB: 6-phosphofructo-2-kinase/fructose-2, 6-bisphosphatase; F-1,6-P_2_: fructose-1, 6-bisphosphatase 2; PK: pyruvate kinase; PI3K/Akt: phosphoinositide 3-kinase/protein kinase B; VEGF: vascular endothelial growth factor; TCA: tricarboxylic acid; FAO: fatty acid oxidation; ATP: adenosine triphosphate; FAs: fatty acids) [11,12,13,14,15,16,17,18].

### 2.1. Reprogramming of Lipid Metabolism in ECs

Fatty acids (FAs) are one of the fuel sources for ECs. Intracellular lipid droplets (LDs) can be formed and metabolized in ECs. In the aortic ECs of mice following olive oil gavage, LDs become readily detectable under confocal microscopy [19]. Lipids can be assembled, stored, and released by LDs. When there is excess energy, free fatty acids (FFAs) can be esterified to triglycerides (TG), which are stored in LDs.

Firstly, according to recent studies, the differences in lipid metabolism of ECs are related to the regions where they are located. A single-cell analysis of the aortas of wild type mice showed that there are three different subpopulations of ECs. The largest subpopulation (EC1) expresses the typical EC marker vascular cell adhesion molecule 1(VCAM1), as well as other genes with known functions such as clusterin (Clu), gastrokine3 (Gkn3), and cytokine-like 1 (Cytl1). The second population (EC2) expresses genes involved in lipid transport, such as cluster of differentiation 36 (CD36), fatty acid-binding protein 4 (Fabp4), lipoprotein lipase (LPL), and glycosylphosphatidylinositol-anchored high-density lipoprotein (HDL)-binding protein 1 (GPIHBP1) and vascular endothelial growth factor (VEGF) receptor Flt1 (VEGFR1). The specific expression differences between the two EC subpopulations still exist after exposure to a high-fat diet. In addition to differences in expression, EC1 (VCAM1) and EC2 (CD36) have unique spatial distributions. The VCAM1/CD36 ratio is lower in the large curvature region of the aortic arch, where atherosclerosis is relatively less likely to occur. But higher scavenger receptor class B type 1 (SR-B1) expression is found in the small curvature region, where atherosclerosis is more likely to occur. Variations in shear stress could be the cause of this [20].

While large-vessel ECs exhibit regional metabolic specialization, ECs in different vascular beds also perform distinct functions. Capillary and venular ECs are primarily responsible for nutrient exchange, whereas aortic and arterial ECs are critical for maintaining hemodynamic homeostasis and represent primary sites of clinical atherogenesis [21]. In the capillary endothelium, lipid uptake begins in the form of FFAs, during which FFAs enter endothelial cells via CD36 and fatty acid transport proteins (FATPs), and GPIHBP1 transports LPL from the subendothelium to the luminal side of ECs. In order to attach to Coenzyme A (CoA), FFAs enter the cell using adenosine triphosphate (ATP) generated by mitochondria. Finally, they are utilized for fatty acid oxidation (FAO), TG synthesis, LDs formation, or transporting out of the cell. Diacylglycerol acyltransferase 1 (DGAT1) and adipose triglyceride lipase (ATGL) are the rate-limiting steps for TG synthesis and hydrolysis, respectively [19]. Various cell-intrinsic regulators, such as mitochondrial ATP, prohibitin (PHB)/Annexin A2 (ANXA2), and mesoderm homeobox (Meox2)/transcription factor (Tcf15), control endothelial FAs transport.

Beyond physiological regulation, disruption of lipid metabolic pathways can directly promote endothelial dysfunction and atherosclerosis. According to recent studies, after high-fat feeding, endothelium-specific ATGL-deficient mice led to lipid accumulation in the vasculature and accelerated the development of atherosclerosis, and ATGL deficiency caused endothelial activation induced by endoplasmic reticulum stress through nuclear factor-κB (NF-κB) signaling [22]. In human aortic ECs, ATGL knockdown promotes the adhesion of monocytes to activated ECs through NF-κB and protein kinase C(PKC)-dependent upregulation of intercellular cell adhesion molecule-1(ICAM-1) [23]. Therefore, ATGL-targeted therapy may be the next new direction for the treatment of atherosclerosis. In addition to traditional lipid uptake routes, recent evidence suggests alternative mechanisms for lipoprotein particle internalization in large vessels. Emerging evidence suggests that in large arteries, chylomicrons (CM) may be absorbed via mechanisms independent of CD36. CM may interact with the endothelial cell surface and be internalized through macropinocytosis upon binding to specific glycoproteins. Alternatively, like LDL and HDL, the uptake of CM may be mediated by specific lipoprotein receptors through cytosis, according to studies of subpopulations of aortic ECs [24]. The severity of atherosclerosis is exacerbated in iLpl^−/−^/Ldlrk^−/−^ mice with hyperchylomicronemia. This suggests that prolonged exposure of VSMCs to chylomicrons and their remnants can potentiate inflammatory responses, likely through the upregulation of adhesion molecules such as VCAM-1, thereby promoting monocyte adhesion [25]. LPL deficiency is closely associated with familial chylomicronemia syndrome. Both conditions can lead to the development of severe hypertriglyceridemia. Therefore, beyond their role in lipid transport, chylomicrons and their remnants directly contribute to endothelial inflammation and atherogenesis. Consequently, therapeutic strategies aimed at reducing circulating levels of CM remnants or attenuating their pro-inflammatory effects on the endothelium represent promising avenues for combating atherosclerosis.

Following the uptake of lipids, ECs within atherosclerotic regions undergo significant reprogramming of intracellular cholesterol metabolism. This dysregulation disrupts cellular homeostasis, amplifies inflammatory responses, and creates a vicious cycle that perpetuates endothelial dysfunction and drives plaque progression [13]. The symbol of this metabolic shift is the imbalance between cholesterol influx and efflux. While ECs express receptors like lectin-like oxidized low-density lipoprotein receptor 1 (LOX-1) and SR-B1 for cholesterol uptake, their efflux capacity, primarily mediated by ATP-binding cassette transporters A1 and G1 (ABCA1 and ABCG1), is often fully loaded under atherosclerotic conditions. Pro-inflammatory cytokines and disturbed flow downregulate these efflux transporters, leading to intracellular cholesterol accumulation [14]. This accumulated cholesterol is esterified by acyl-CoA cholesterol acyltransferase (ACAT) and stored in LDs, or remains unesterified in the cytoplasm, where it can disrupt the tunica media fluidity and trigger endoplasmic reticulum (ER) stress [13]. The consequences of this cholesterol contribute to a maladaptive cycle of endothelial activation. Excess intracellular cholesterol inhibits the activity of eNOS and contributes to mitochondrial dysfunction, leading to diminished NO production and increased generation of ROS. This impairs vasodilation and amplifies oxidative stress. Unesterified cholesterol can activate the NOD-like receptor thermal protein domain-associated protein 3 (NLRP3) inflammasome, resulting in the cleavage and secretion of interleukin-1β (IL-1β) [15]. Furthermore, cholesterol accumulation activates NF-κB signaling, promoting the expression of adhesion molecules like VCAM-1 and ICAM-1, which facilitate monocyte recruitment and adhesion [26]. Cholesterol-induced inflammation and oxidative stress damage endothelial barrier integrity, increasing vascular permeability. This allows further infiltration of circulating lipids and immune cells into the tunica intima, developing lesion development [14].

In brief, the dysregulated cholesterol metabolism establishes a pathological feedback loop. Inflammation and oxidative stress induced by the disorder of cholesterol metabolism further suppress cholesterol efflux pathways and enhance pro-inflammatory signaling, continuously driving endothelial activation and creating a microenvironment conducive to atherosclerosis progression.

### 2.2. Reprogramming of Glucose Metabolism in ECs

ECs exhibit a unique bioenergetic strategy known as aerobic glycolysis or the Warburg effect. Its characteristic is to metabolize at a high flux of glycolysis, even in the presence of ample oxygen, rather than relying primarily on oxidative phosphorylation for ATP generation. Katrien et al. measured the flux of metabolic pathways and discovered that arteriolar, lymphatic, and microvascular ECs had a high degree of glycolysis. This level of glycolysis was almost identical to that of tumor cells and significantly higher than that of other types of healthy cells. Glycolytic fluxes were more than 200 times greater than those of glucose oxidation, fatty acid oxidation, and glutamine oxidation. Therefore, it is thought that the primary energy supply channel for ECs is glycolysis [16,17]. This metabolic reprogramming is exacerbated in disease. For example, a 2016 study found that atherosclerotic plaques from apolipoprotein E (ApoE)-deficient mice had much higher glucose uptake and glycolytic rates than those from wild-type mice, highlighting a pathologically enhanced glycolytic phenotype in the atherosclerotic microenvironment [27]. Through a series of inflammatory reactions, injured ECs cause the development of atherosclerosis during the pre-atherosclerosis phase.

Aerobic glycolysis is governed by three key rate-limiting enzymes: hexokinase (HK), phosphofructokinase-1 (PFK-1), and pyruvate kinase (PK). HK, the first key enzyme, catalyzes the phosphorylation of glucose to glucose-6-phosphate (G6P), trapping it within the cell for glycolytic processing. Beyond its role in energy production, HK can promote cell proliferation and exert anti-apoptotic effects. Notably, HK binding to mitochondrial voltage-dependent anion channels (VDAC) prevents the recruitment of pro-apoptotic proteins like Bax, thereby enhancing cell survival [28,29].

PFK-1 is the second rate-limiting enzyme of glycolysis. F2, 6BP is the most powerful of the variables that regulate PFK-1 expression, while 6-phosphofructo-2-kinase/fructose-2, 6-bisphosphatase (PFKFB) controls the amount of F2, 6BP. PFKFB3 is a bifunctional enzyme that is a member of the 6-phosphofructo-2-kinase/phosphofructokinase-2 (PFK-2) family. It has the bisphosphatase structural domain fructose-2, 6-bisphosphatase 2 (FBPase-2), as well as the kinase structural domain PFK-2. In order to regulate the in vivo concentration of F2, 6BP to reach homeostasis, PFKFB3 catalyzes its synthesis via PFK-2 and its breakdown via FBPase-2. F2, 6BP is not only an activator of PFK-1 but also an inhibitor of fructose 1, 6-bisphosphatase-1(FBPase-1), a key enzyme in gluconeogenesis [18]. Overall, PFKFB3 greatly accelerates the glycolytic flux of PFK-1, which could upregulate the glycolysis of ECs, promote the proliferation and migration of ECs and ultimately trigger angiogenesis. VEGF potently stimulates ECs migration, a process that demands substantial energy for cytoskeletal remodeling. To meet this surge in energy demand, VEGF signaling markedly upregulates the glycolytic rate in ECs. This is primarily achieved through the upregulation of 6-phosphofructo-2-kinase/fructose-2,6-bisphosphatase 3 (PFKFB3), the enzyme that synthesizes fructose-2,6-bisphosphate (F2, 6BP). F2, 6BP is a potent allosteric activator of PFK-1, the key rate-limiting enzyme of glycolysis, thereby pushing glycolytic flux to meet migratory demands [16]. However, the flow-responsive transcription factor Krüppel-like factor 2 (KLF2) has been shown to bind to the PFKFB3 promoter and inhibit its transcription [26], suggesting a mechanism for metabolic regulation under shear stress conditions. By suppressing PFKFB3 transcription, KLF2 ultimately leads to a reduction in glycolytic flux, thereby antagonizing the VEGF-induced metabolic switch and contributing to the maintenance of endothelial quiescence [30]. The activity of PFKFB3 is regulated by multiple signaling pathways. While the rat sarcoma (RAS) pathway can influence its kinase activity, the Adenosine 5’-monophosphate-activated protein kinase (AMPK) plays a context-dependent role. In ECs under hemodynamic stress, AMPKα1 activation can promote a compensatory increase in glycolysis by upregulating hypoxia-inducible factor-1α (HIF-1α) and the transcription of glycolytic enzymes, which supports cell survival and proliferation. Consistently, endothelial-specific knockdown of AMPKα1 was shown to decrease glycolytic flux, reduce the proliferation of ECs, increase vascular permeability, and accelerate atherosclerotic lesion formation in mice [31]. This demonstrates that AMPKα1-mediated glycolysis can be atheroprotective in certain contexts. Enhancement of ECs glycolysis by overexpression of the glucose transporter 1(GLUT1) via adenoviral vectors can attenuate the down-regulation of glycolysis due to AMPKα1 deletion, enhance the activity of ECs, protect endothelial integrity, and finally reverse the susceptibility to the development of atherosclerosis [32]. In addition, PFKFB3 promotes ECs inflammation via tumor necrosis factor-α(TNF-α), which promotes the development of atherosclerosis [12]. This illustrates the “double-edged sword” nature of endothelial glycolysis, which can be either adaptive or maladaptive depending on the pathophysiological setting.

As the third essential enzyme in glycolysis, PK catalyzes the transformation of phosphoenolpyruvate into pyruvate and the subsequent synthesis of ATP. PK consists of two isozymes, M-type and L-type. M-type contains the M1 and M2 isoforms. Pyruvate kinase isozyme type M2 (PKM2) is the only PK isoform that alternates between a low-active dimeric form and a highly active tetrameric form. PKM2 undergoes phosphorylation to change into its dimeric form, facilitates the entry of glycolytic products upstream of PK into the biosynthetic pathway, and synthesizes the macromolecules the body needs [33]. PKM2 expression was found to be regulated by the phosphoinositide 3-kinase/protein kinase B (PI3K/Akt) signaling pathway [11]. Beyond ECs, PKM2 plays a pro-atherogenic role in other key cells. In VSMCs, PKM2-driven glycolysis facilitates the phenotypic switching, proliferation, and migration that contribute to new tunica intima formation. Similarly, PKM2 is upregulated in monocytes and macrophages within human atherosclerotic plaques, where it modulates inflammatory responses and foam cell formation—aspects that will be elaborated in the subsequent sections on VSMCs and macrophages.

In addition to protein-level regulation, glycolysis is also modulated at the post-transcriptional level by microRNAs, which have been reported to significantly influence glycolytic flux and endothelial cell function in atherosclerosis. The microRNA miR-143 has emerged as a critical negative regulator of glycolysis in atherosclerosis. While glycolytic rates are high and miR-143 levels are low in healthy endothelium, miR-143 is upregulated in atherosclerotic plaques. It directly targets the mRNA of key glycolytic enzymes, including HK2, lactate dehydrogenase A (LDHA), and PKM2, leading to their downregulation. This miR-143-mediated suppression of glycolysis contributes to endothelial dysfunction [34], and conversely, strategies to enhance glycolysis can ameliorate it. Additionally, it shows that increased glycolysis can alleviate the dysfunction of ECs. The role of AMPK in ECs glycolysis is context-dependent. In hemodynamically disturbed endothelium, protein Kinase AMP-Activated Catalytic Subunit Alpha 1 (PRKAA1)/AMPKα1 expression is elevated. In this setting, AMPK can promote the transcription of glycolytic enzymes via upregulation of HIF-1α. This AMPK/HIF-1α-driven increase in glycolysis supports ECs proliferation and has been shown to slow the progression of atherosclerosis in certain models, highlighting the complex and sometimes paradoxical role of metabolic signaling in disease [32].

The role of glycolysis in atherosclerosis exemplifies that a balanced level is crucial for maintaining endothelial homeostasis and function, but both deficiency and excess are detrimental. While augmented glycolysis can rescue endothelial dysfunction under stress, excessive glycolytic flux drives pathological endothelial hyperproliferation and compromises barrier integrity, thereby accelerating the progression of atherosclerosis [35]. This dual nature suggests that therapeutic strategies must be finely tuned. Future therapies may need to target specific glycolytic nodes in a cell-type and context-dependent manner to restore metabolic equilibrium, representing a novel and promising approach for treating atherosclerosis.

Glucose diverts into two side-branch pathways: the hexosamine biosynthesis pathway (HBP) and the pentose phosphate pathway (PPP). These pathways serve distinct biosynthetic and redox functions beyond ATP production. The HBP feeds on glucose, glutamine, acetyl coenzyme A, and uridine to produce N-acetylglucosamine, which is used for protein O-glycosylation and N-glycosylation [36]. The PPP, initiated from G6P, fulfills two primary roles: it generates ribose-5-phosphate and nicotinamide adenine dinucleotide phosphate (NADPH). NADPH is a critical reducing equivalent. It fuels antioxidant systems and supports reductive biosynthesis for maintaining redox homeostasis. Consequently, inhibition of key PPP enzymes not only impairs nucleotide synthesis but also induces oxidative stress, leading to diminished the migration and viability of ECs [37].

One of the most significant outcomes of enhanced glycolysis is the accumulation of lactate, which is now recognized as far more than a metabolic waste product. Lactate is a crucial signaling molecule that activates signaling pathways in ECs and regulates inflammatory responses. It is primarily produced by glycolysis. Importantly, in inflammatory cells within plaque, lactate is generated in large quantities even under adequate oxygen conditions. Independent of other conventional cardiovascular risk factors, patients with established atherosclerosis have been found to have higher blood lactate levels, indicating that lactate plays a role in the onset and progression of atherosclerosis [38]. Hypoxia and inflammation in atherosclerosis increase the production and release of lactate and encourage both anaerobic and aerobic glycolysis. Through the activation of ECs in both HIF-dependent and HIF-independent mechanisms, lactate can stimulate angiogenesis. Lactate enters ECs by monocarboxylate transporter 1(MCT1) in the HIF-independent pathway, where it triggers angiogenesis in a way that is dependent on ROS and NF-κB/interleukin-8 (IL-8). Lactate also enters ECs through MCT1 in the HIF-dependent pathway, which inactivates prolyl hydroxylase (PHD), stabilizes HIF-1α, and transcriptionally induces VEGF production to support angiogenesis [39,40]. Angiogenesis is a significant pathogenic characteristic of atherosclerotic lesions, and neovascularization can either worsen atherosclerotic plaque instability or encourage plaque remodeling and development. For instance, a considerable degree of plaque neovascularization has been noted in patients with diabetic carotid atherosclerotic plaques. As a result, it is believed that neovascularization may play a role in the development of susceptible plaques in advanced atherosclerotic lesions [41]. Conversely, lactate can also exert atheroprotective effects by acting as an agonist for the G protein-coupled receptor 81 (GPR81). In ECs exposed to physiological laminar shear stress, lactate upregulates GPR81 expression, and its binding activates a signaling cascade that promotes the expression of the protective transcription factor KLF2. KLF2 suppresses pro-inflammatory endothelial activation and helps maintain vascular homeostasis [42].

The interplay between metabolic rewiring and inflammation is a hallmark of atherosclerosis. Altered metabolite levels, such as lactate, can directly influence the production and response of cytokines, which in turn are central drivers of disease progression or protection. Cytokines play divergent roles in the pathogenesis of atherosclerosis, broadly categorized as pro-inflammatory and anti-inflammatory. Key pro-inflammatory cytokines, such as TNF-α and interleukin-1 (IL-1), are often upregulated via NF-κB signaling and drive endothelial dysfunction, leukocyte recruitment, and plaque inflammation. Conversely, cytokines like interleukin-10 (IL-10) and transforming growth factor-β (TGF-β) are generally considered anti-inflammatory and can exert atheroprotective effects [43,44]. To systematically explore the interface between glycolysis and inflammation in atherosclerosis, Wang et al. employed bioinformatic analyses of the NCBI database, protein–protein interaction networks, and Cytoscape(Cytoscape 3.2.1 software). Their predictive analysis identified several hub targets linking these processes, including Akt1, interleukin-6 (IL-6), VEGFA, tumor protein 53 (TP53), Signal Transducer And Activator Of Transcription 3 (STAT3), Steroid receptor coactivator (Src), and Mitogen-Activated Protein Kinase 1 (MAPK1). Notably, this network integrates key glycolytic regulators like PFKFB3 and lactate transporters like MCT1 with established inflammatory and signaling pathways, providing a computational framework and promising targets for future mechanistic and therapeutic research in atherosclerosis [45]. Thus, lactate also acts as a double-edged sword in atherosclerosis. Its net effect likely depends on the concentration, metabolic context, and the local cellular microenvironment, determining whether it drives pathological angiogenesis or promotes anti-inflammatory protection.

### 2.3. Endothelial Metabolic Crosstalk in Atherosclerosis

The glycose and lipid metabolic reprogramming of ECs in atherosclerosis represents a core pathological mechanism linking metabolic dysregulation, inflammatory response, and vascular dysfunction. In terms of lipid metabolism, the process is primarily characterized by cholesterol accumulation resulting from increased influx and impaired efflux, which subsequently induces endoplasmic reticulum stress, oxidative stress, and a potent inflammatory response. Regarding glucose metabolism, there is a marked enhancement of glycolytic flux, mediated by key enzymes such as PFKFB3 and metabolites like lactate, which exert dual roles in modulating endothelial inflammation, proliferation, and barrier function. Notably, glucose and lipid metabolic disturbances are not isolated events but interact closely through signaling molecules such as ROS, eNOS, and NF-κB, forming a vicious cycle that amplifies pathological progression. This metabolic rewiring not only elucidates a novel mechanism in atherogenesis but also reveals potential therapeutic windows for targeting metabolic pathways—such as PFKFB3, ATGL, lactate receptors, or cholesterol transporters.

## 3. Glucose and Lipid Metabolism of Macrophages in Atherosclerosis

Macrophage metabolic reprogramming and polarization are critically implicated in every phase of atheromatous plaque evolution, including initiation, progression, rupture, and subsequent healing.

### 3.1. Macrophage Polarization States and Subpopulations

The alteration of macrophage functional phenotype essentially depends on their activation state or their response to environmental stimuli. The macrophage subtypes currently identified include M1, M2, M (Hb), Mhem, Mox, and M4. The diversity of these subsets reflects the complex microenvironment of the advancing plaque, with recent single-cell studies further revealing a continuum of macrophage states [46]. However, most studies still use the M1/M2 classification to summarize their characteristics.

This polarization is underpinned by distinct metabolic programs, wherein M1 macrophages rely on aerobic glycolysis, while M2 polarization is supported by oxidative metabolism [47].In the early stages of the lesion, the plaque environment is enriched with M2 macrophages that confer structural stability through abundant collagen secretion and efficient efferocytosis. However, as the lesions progress, this anti-inflammatory profile is progressively lost. The number of M2 macrophages decreases, while classically activated M1 macrophages accumulate in large numbers and enhance the release of proinflammatory cytokines, thereby increasing the risk of plaque rupture. Concomitantly, expression of the cholesterol-efflux transporter ABCA1 diminishes, impeding cholesterol export and promoting intracellular lipid retention. The resulting excess cholesterol further fuels macrophage activation and sustains M1 polarization, establishing a self-perpetuating pathogenic loop [48,49,50,51].

### 3.2. Reprogramming of Lipid Metabolism in Macrophages

Fatty acids are oxidized in the mitochondria to generate acetyl coenzyme A (acetyl-CoA), which enters the tricarboxylic acid (TCA) cycle for complete oxidation and efficient ATP production. Conversely, de novo fatty acid synthesis occurs under conditions of sufficient intracellular NADPH availability, with fatty acid synthase (FAS) acting as the rate-limiting enzyme. Inflammatory M1 macrophages exhibit enhanced FAS expression and lipogenesis, whereas M2 macrophages maintain an intact TCA cycle and rely predominantly on FAO to support their oxidative metabolic phenotype [52]. FAS deficiency alters plasma membrane composition and disrupts ras homology (Rho) GTPase trafficking, thereby attenuating pro-inflammatory signaling in macrophages [53]. Additionally, specific oxysterols and fatty acid derivatives can activate peroxisome proliferator-activated receptors (PPARα and PPARγ), which suppress NF-κB signaling and may promote an anti-inflammatory M2-like polarization state [54]. While the balance between fatty acid synthesis and oxidation significantly influences macrophage polarization, recent studies highlight a more complex interplay between these metabolic pathways and inflammatory outcomes. Emerging evidence indicates that FAO contributes not only to M2 polarization, but also to NLRP3 inflammasome activation in M1 macrophages. Furthermore, glycolytic flux provides essential TCA cycle intermediates that support FAO in M2 macrophages, illustrating the interconnectivity between glucose and lipid metabolism [55].

Beyond fatty acid metabolism, cholesterol homeostasis is equally critical in determining macrophage fate and function in atherosclerosis. Imbalance in cholesterol uptake and efflux drives foam cell formation, a hallmark of early atherosclerotic lesions. The LOX-1 is upregulated by inflammatory signals, advanced glycation end products (AGEs), and ox-LDL, increasing lipid uptake in macrophages [56,57]. Cholesterol efflux is mediated by ABCA1, ABCG1, and SR-B1. When the expression of ABCA1 and ABCG1 is decreased, the buildup of cholesterol in macrophages encourages the formation of foam cells [58]. PKM2 deletion in macrophages raises the production of the LDL-related protein-1 (LRP-1), which may slow the course of atherosclerosis by modifying atherosclerotic microenvironmental inflammation. The exact mechanism of this response is still under investigation [59]. PKM2 triggers STAT3, which increases the inflammatory response by promoting the transcription of the pro-inflammatory genes IL-6 and IL-1β. It facilitates NLRP3 inflammasome assembly, leading to IL-18/IL-1β release and VSMC proliferation. In general, PKM2-driven glycolysis increases plaque vulnerability [60,61]. MiRNAs play critical roles in regulating macrophage lipid metabolism during atherosclerosis. For instance, miR-33 targets ABCA1 to suppress cholesterol efflux, promoting foam cell formation. Conversely, inhibition of miR-33 enhances ABCA1 expression and stimulates reverse cholesterol transport [62]. Additionally, miR-27b suppresses PPARγ and LPL expression, altering fatty acid uptake and storage [63]. These miRNA-mediated mechanisms intricately modulate lipid homeostasis in macrophages and represent potential therapeutic targets for atherosclerotic lesions.

### 3.3. Reprogramming of Glucose Metabolism in Macrophages

**Figure 2:**
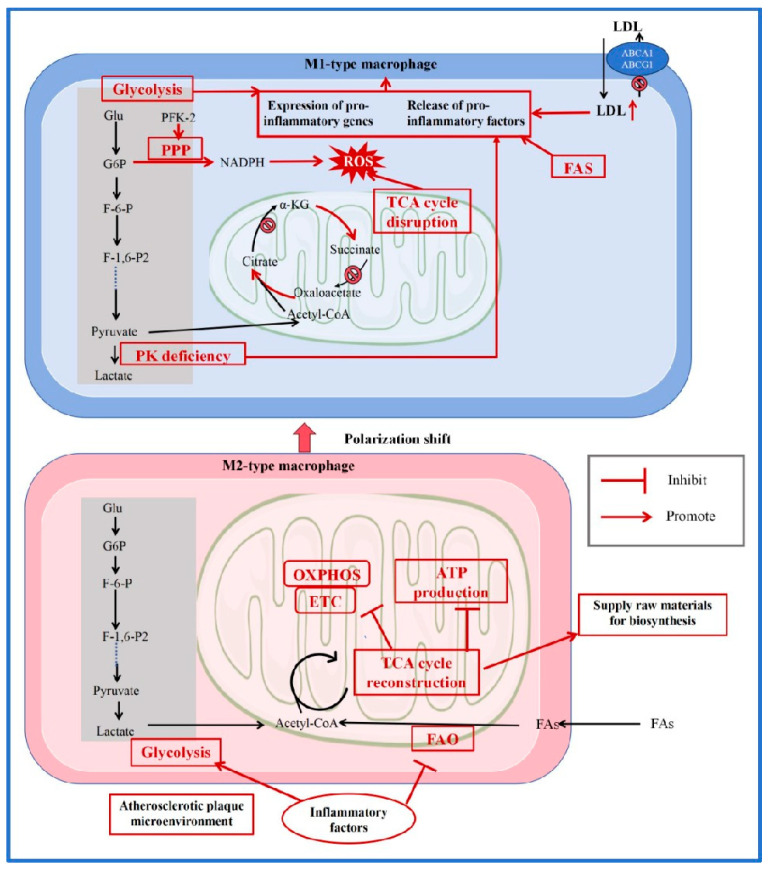
Reprogramming of Glucose and Lipid Metabolism in Macrophages.

Metabolic reprogramming is a key determinant of macrophage function. In atherosclerosis, both extrinsic (e.g., cytokines, lipids) and intrinsic factors drive metabolic alterations in macrophages, affecting their polarization and function within the plaque microenvironment [64]. Macrophages are dynamic and change in plasticity and heterogeneity as they undergo metabolic reprogramming in the microenvironment, and they also have an impact on the plaque microenvironment. We have provided a basic introduction to the metabolic reprogramming and polarization conversion of macrophages in Figure 2.

In the atherosclerotic lesion area, macrophage polarization tends toward the pro-inflammatory M1 phenotype, prompting a shift in M2-type macrophages toward M1 polarization. In M1 macrophages, accumulated LDL, high expression of FAS, and loss of PK activity collectively promote the release of pro-inflammatory factors. Increased glycolytic flux further enhances the expression of pro-inflammatory genes. PFK-2 activation stimulates the PPP, resulting in increased NADPH production and subsequently elevated ROS. Concurrently, impaired enzyme activities in the TCA cycle and disrupted metabolic pathways also contribute to ROS accumulation, ultimately driving the production and release of inflammatory factors. In contrast to M1 macrophages, M2 macrophages rely more on an intact TCA cycle and oxidative phosphorylation for their functions. Thus, when transitioning toward the M1 phenotype, M2 macrophages undergo metabolic reprogramming—shifting from oxidative phosphorylation and energy production toward a glycolysis-dominated metabolism that supplies biosynthetic precursors. (LDL: Low-Density Lipoprotein; ROS: reactive oxygen species; ABCA1: ATP-binding cassette transporters A1; ABCG1: ATP-binding cassette transporters G1; Glu: glucose; G6P: glucose-6-phosphate; F-6-P: fructose 6-phosphate; PFK-2: phosphofructokinase-2; F-1,6-P2: fructose-1, 6-bisphosphatase 2; PK: pyruvate kinase; TCA: tricarboxylic acid; FAO: fatty acid oxidation; ATP: adenosine triphosphate; FAs: fatty acids; PPP: pentose phosphate pathway; FAS: fatty acid synthase; NADPH: nicotinamide adenine dinucleotide phosphate; OXPHOS: oxidative phosphorylation; ETC: electron transfer chain; α-KG: α-ketoglutarate; acetyl-CoA: acetyl coenzyme A) [64,65,66,67,68,69].

Glycolysis is markedly enhanced in classically activated M1 macrophages. Inflammatory stimuli like interferon-γ (IFN-γ) upregulate specific isoforms of PFK-2, accelerating glycolytic flux [68,69]. This enables rapid ATP generation and supports biosynthetic pathways. Concurrently, the TCA cycle is disrupted, leading to accumulation of metabolites such as citrate and succinate. Citrate supports NO production, while succinate stabilizes HIF-1α, further amplifying glycolytic activity and pro-inflammatory gene expression [65,66,67]. The PPP is also activated, generating NADPH for ROS production. This metabolic configuration reinforces and sustains the M1 inflammatory phenotype [70].

In contrast, alternatively activated M2 macrophages maintain an intact TCA cycle and exhibit greater dependence on oxidative phosphorylation. Their metabolism supports anti-inflammatory functions and tissue repair. Inhibition of HIF-1α has been shown to promote M2 polarization, underscoring the reciprocal relationship between metabolic state and phenotype [71].

Lactate, a key glycolytic end product, plays complex roles in macrophage polarization. It can promote M2-like polarization through HIF-1α-dependent and independent mechanisms. Following uptake via monocarboxylate transporters, lactate can enhance expression of M2-associated genes (Arginase-1/Arg1, found in inflammatory zone 1/ Fizz1) via HIF-1α stabilization [72]. Alternatively, it can activate mechanistic target of rapamycin complex 1 (mTORC1) signaling while inhibiting transcription factor EB (TFEB), leading to HIF-2α accumulation and expression of homeostatic genes (mannose receptor C-type 1/Mrc1, Arg1). Lactate also exerts anti-inflammatory effects by several mechanisms: it impairs TNF-α secretion under acidic conditions, suppresses NF-κB activity via GPR81 signaling, and inhibits NLRP3 inflammasome activation. Further modulation occurs through G protein-coupled receptor 65 (GPR65)-mediated suppression of inflammatory gene expression [73,74,75,76,77].

Beyond lactate, methylglyoxal (MGO) represents another significant glycolytic derivative that leads to atherosclerosis. This highly reactive dicarbonyl compound rapidly modifies proteins to form AGEs. Macrophages that accumulate AGEs exhibit increased apoptosis, a process that contributes to necrotic core expansion and plaque destabilization. However, the detailed mechanisms underlying this pathway require further investigation [78]. Thus, glucose metabolism actively shapes macrophage behavior in atherosclerosis through multiple pathways and metabolites, influencing both polarization status and inflammatory output.

### 3.4. Summary of Macrophage Metabolic Reprogramming and Polarization in Atherosclerosis

Macrophage metabolic reprogramming drives phenotypic polarization and plaque progression in atherosclerosis. Pro-inflammatory M1 macrophages rely on glycolysis and impaired cholesterol efflux, promoting lipid accumulation and inflammation. In contrast, M2 macrophages utilize oxidative metabolism to support anti-inflammatory functions. Key metabolites such as lactate exert dual roles by modulating inflammatory signaling through receptors including GPR81. Targeting metabolic checkpoints—such as PFKFB3, PKM2, miR-33, or lactate-related pathways—may restore metabolic balance, suppress M1 polarization, and promote plaque stability, offering promising therapeutic strategies for atherosclerosis.

## 4. Metabolic Reprogramming of VSMCs in Atherosclerosis

VSMCs are central contributors to atherosclerotic lesion development and stability. They can exert dual roles: forming a protective fibrous cap over the necrotic core through extracellular matrix (ECM) production, or migrating into early lesions and accelerating plaque progression. During atherosclerosis, VSMCs undergo a phenotypic switch from a contractile to a synthetic state, enabling proliferation, migration, and macromolecular biosynthesis to meet heightened metabolic demands [79,80]. In vivo, VSMCs can also exhibit a variety of alternate phenotypes, such as macrophage-like, foam cell-like, osteochondrogenic-like, myofibroblast-like, and mesenchymal-like VSMCs [81]. Emerging evidence indicates that metabolic reprogramming modulates VSMC plasticity in atherosclerosis. Quiescent contractile VSMCs primarily rely on oxidative phosphorylation for energy production, whereas synthetic VSMCs shift toward glycolysis to support biosynthetic and migratory activities [82].

In healthy arteries, VSMCs maintain a contractile phenotype characterized by high expression of markers such as myosin heavy chain 11 (MYH11), transgelin (TAGLN), and smooth muscle α-actin 2 (ACTA2) [83]. Contractile marker genes in VSMCs are down-regulated during vascular injury [84]. Key regulators of VSMC contractile gene expression include serum response factor (SRF) and its coactivator myocardin (MYOCD). SRF depends on MYOCD to activate transcription of contractile genes [85]. The phenotypic transition of VSMCs is regulated by signaling pathways involving growth factors such as platelet-derived growth factor (PDGF-BB) and TGF-β, transcription factors such as NF-κB, octamer-binding transcription factor 4 (OCT4) and KLF4, as well as epigenetic modifiers [81]. Phenotypically modulated VSMCs secrete numerous ECM components and pro-inflammatory mediators. VSMC-derived ECM constitutes a major structural component of the vascular wall and plaque cap, with its turnover regulated by a dynamic balance between synthesis and degradation. In early atherosclerosis, synthetic VSMCs, macrophage-like VSMCs, and VSMC-derived foam cells produce matrix metalloproteinases (MMPs), which degrade pericellular connective tissue, facilitate VSMC migration, and promote plaque formation and remodeling [86]. A recent proteomic study identified a 4-biomarker signature—comprising MMPs, S100A8/S100A9, histone D, and galectin-3-binding protein—in ECM-derived fractions that distinguishes symptomatic from asymptomatic carotid plaques, highlighting the clinical relevance of VSMC-mediated matrix remodeling [87].

Although VSMCs can exert vasoprotective effects through ECM secretion, their overall impact in atherosclerosis may be detrimental due to sustained release of pro-inflammatory mediators [88]. Synthetic VSMCs secrete cytokines such as IL-1β, IL-6, and monocyte chemoattractant protein-1 (MCP-1), which promote monocyte recruitment and accelerate atherosclerotic plaque development [89]. Additionally, VSMC-derived extracellular vesicles (EVs) play a key role in pathological intercellular communication. These EVs carry bioactive cargo—including lipids, proteins, nucleic acids, and microRNAs—that can reprogram recipient cells and promote processes such as vascular calcification [90]. Senescent VSMCs further contribute to plaque progression through the senescence-associated secretory phenotype (SASP), characterized by sustained release of inflammatory factors that exacerbate local inflammation and drive phenotypic switching of neighboring VSMCs [91]. Metabolic reprogramming is increasingly recognized as a key regulator of VSMC behavior. PDGF stimulation enhances glycolytic flux in VSMCs, as evidenced by increased glucose uptake and elevated lactate production. LDHA, which catalyzes the conversion of pyruvate to lactate, plays a critical role in supporting VSMC proliferation and migration [92]. Suppression of LDHA attenuates glycolytic flux and reduces VSMC motility and growth. Moreover, lactate promotes vascular calcification by upregulating nuclear receptor 4A1 (NR4A1) expression, leading to DNA-dependent protein kinase catalytic subunit (DNA-PKcs) and p53 activation, enhanced mitochondrial fission, impaired mitophagy, and ultimately osteogenic differentiation of VSMCs [93]. Ox-LDL also enhances glycolytic activity in VSMCs in a PKM2-dependent manner, further promoting proliferation and migration [35]. These findings highlight aerobic glycolysis as a promising therapeutic target for attenuating VSMC-driven atherosclerosis.

VSMCs undergo a phenotypic transition from a contractile to a synthetic state during atherosclerosis, accompanied by a metabolic shift from OXPHOS toward glycolysis. This reprogramming supports proliferation, migration, and inflammatory mediator secretion, but also contributes to plaque instability through enhanced extracellular matrix remodeling and osteogenic differentiation. Targeting metabolic nodes such as LDHA, PKM2, or lactate signaling may offer novel strategies to modulate VSMC plasticity, maintain plaque stability, and ameliorate atherosclerotic progression.

## 5. Intercellular Metabolic Cross-Talk in Atherosclerosis

Research exploring the molecular mechanisms of metabolic dysregulation in atherosclerosis primarily relies on two experimental paradigms: (i) using purified cell types in vitro and (ii) genetic knockout of essential metabolic enzymes in specific cell subpopulations in vivo. However, these approaches may overlook the critical intercellular communication that integrates multiple cell types and enzymatic networks, potentially neglecting clinically relevant interactions that occur during the development of human atherosclerosis. Additionally, metabolites can function as signaling molecules between diseased cells. Their soluble products extend metabolic effects to distant cell populations, thereby determining cell fate and functional programming.

Oxycholesterol, as a cholesterol metabolite, plays multiple roles in lipid metabolism. 27-Hydroxycholesterol (27HC) is one of the primary oxidative derivatives of cholesterol. The synthesis of 27-hydroxycholesterol is catalyzed by the enzyme sterol 27-hydroxylase (CYP27A1), while its metabolism is handled by oxysterol 7α-hydroxylase (CYP7B1). Studies have shown that elevated levels of 27HC resulting from CYP7B1 deficiency attenuate estrogen-mediated atheroprotection [94]. In wild-type mice, 27HC enhances leukocyte-endothelial adhesion through estrogen receptor (ER)-dependent mechanisms. In monocytes/macrophages, 27HC upregulates pro-inflammatory genes and promotes adhesion specifically via ERα. Similarly, in ECs, 27HC increases adhesiveness through ERα. However, in contrast to estrogen, which suppresses NF-κB activation, 27HC stimulates NF-κB signaling via extracellular signal-regulated kinase (Erk1/2)- and c-Jun N-terminal kinase (JNK)-mediated degradation of inhibitor of kappa B alpha (IκBα). CYP27A1 is highly expressed in the liver and constitutively present in normal arterial walls, with further upregulation observed in atherosclerotic lesions. Recent evidence indicates that 27HC mediates crosstalk between macrophages and ECs [95]. The pro-atherogenic actions of macrophage-derived 27HC require ERα and disassociation of the cytoplasmic scaffolding protein septin 11 from ERα, leading to extranuclear ERα and septin 11-dependent activation of NF-κB. In parallel, NF-κB activation by septin 11 in endothelium is implicated in the disease-promoting macrophage-to-ECs communication by 27HC. Glycolysis promotes atherosclerosis by driving ECs activation and VSMCs dedifferentiation [32]. However, glycolysis also exerts anti-atherosclerotic effects. It enhances the phagocytic capacity of phagocytes and triggers macrophages to produce pro-lytic mediators [96]. Thus, therapeutic strategies targeting only cholesterol or other individual pathways may disrupt essential intercellular communication, potentially introducing unforeseen risks in the treatment of atherosclerosis.

Additionally, lactate—a glycolytic end product—has emerged as a key mediator of intercellular metabolic crosstalk within atherosclerotic plaques. Macrophages, ECs, and vascular smooth muscle cells can both produce and utilize lactate, creating the inflammatory and metabolic microenvironment of the lesion. For instance, lactate derived from glycolytic macrophages can enhance NF-κB signaling and stabilize HIF-1α in vascular cells, perpetuating a pro-inflammatory state [72]. Meanwhile, lactate uptake by ECs may impair barrier function and promote leukocyte adhesion [97]. In VSMCs, lactate has been shown to induce phenotypic switching and exacerbate calcification [98]. This lactate travels between different cell types, which underscores the complexity of metabolic interactions in atherosclerosis and suggests that targeting lactate metabolism or signaling might offer novel therapeutic opportunities.

## 6. Pharmacologic Treatment of Atherosclerosis and New Advances

### 6.1. Current Therapeutic Strategies for Atherosclerosis

The existing treatment options for atherosclerosis are summarized in Table 1. Emerging therapeutic approaches for atherosclerosis increasingly focus on targeting metabolic pathways central to disease progression. Clinically, low-density lipoprotein cholesterol (LDL-C) is often used as the lipid detection index and treatment target. Lipid-lowering remains the most fundamental and core step in the treatment of atherosclerosis. Statins, ezetimibe, and proprotein convertase subtilisin/kexin type 9 (PCSK9) inhibitors can reduce LDL-C through cholesterol biosynthesis and uptake pathways. Beyond lipid-centric strategies, antidiabetic agents now play a significant role in modulating vascular metabolism and inflammation. Metformin, GLP-1 receptor agonists, and SGLT-2 inhibitors improve glycemic control while exerting pleiotropic effects on endothelial function, macrophage polarization, and plaque stability via AMPK activation, NLRP3 inflammasome suppression, and oxidative stress reduction. Anti-inflammatory therapies are also being refined to target immunometabolic axes, such as chemokine-driven monocyte recruitment and neutrophil extracellular trap formation. Both are fueled by glycolytic and lipid metabolites. This integrated metabolic perspective highlights the convergence of lipid, glucose, and inflammatory pathways in atherosclerosis and offers new opportunities for combinatory therapies that address the multifaceted metabolic dysregulation underlying disease development and progression [99,100,101,102,103,104,105,106,107,108,109,110,111,112].

### 6.2. New Targets for Targeted Therapies

Researchers now have a better understanding of the interactions among various cell types and factors in atherosclerosis, owing to advances in techniques such as metabolomics and single-cell sequencing. Investigations into glucose and lipid metabolism in vascular smooth muscle cells, macrophages, and ECs within atherosclerotic lesions have facilitated the development of therapeutic strategies targeting metabolites and enzymes involved in these metabolic pathways. Such strategies may also apply to the treatment of atherosclerosis-related comorbidities. These advances have also revitalized interest in the potential utility of established medications, such as edaravone dexborneol, which has demonstrated promising effects in the long-term management of vascular atherosclerotic lesions. The novel atherosclerosis therapies targeting metabolism are summarized in Table 2.

We discussed lipid metabolism and glycolysis in atherosclerosis in the preceding section, and it has been demonstrated that addressing glycolysis may have an impact on how atherosclerosis develops [118]. For instance, in VSMCs, therapeutic inhibition of glycolytic activation can be achieved by miR-638, which targets LDHA to suppress VSMC proliferation and migration [119]. In macrophages, the glycolytic inhibitor 2-deoxyglucose (2DG) specifically targets macrophage glycolysis, thereby reducing the production of the pro-inflammatory cytokine IL-1β and modulating plaque inflammation [66]. Since the previous text described the significant effects of lactate on lipid accumulation, VSMC phenotypic transition, intraplaque angiogenesis, inflammation, and vascular calcification, lactate transport proteins may be a viable target for treatment. However, atherosclerosis and glycolysis interact through a variety of intricate processes, and anti-inflammatory treatment cannot be replaced by inhibiting the glycolysis of ECs [32]. Therefore, approaches to inhibit glycolysis therapy still need to be approached with caution. In addition, new targets for lipid-lowering therapies have been proposed, such as cholesteryl ester transfer protein inhibitors and recombinant HDL infusion to raise HDL-C, intestinal microbiome therapies, clonogenic hematopoiesis, targeted proinflammatory cascade response therapies for inflammation, and omega-3 fatty acids and antisense oligonucleotides targeting APOC3 to lower TRL [120]. After being confirmed by additional research, these might eventually be included in statin-based lipid-lowering treatment.

## 7. Conclusions

Atherosclerosis is characterized by the accumulation of lipids, immune cells, and an extracellular matrix, leading to the formation of plaques that can restrict blood flow or finally cause the rupture of atherosclerotic plaques. VSMCs, ECs, and macrophages are important players in this process.

Endothelial cell dysfunction is the starting point of atherosclerosis. Damaged ECs release numerous cytokines and chemokines that promote monocyte adhesion, migration, and differentiation. Furthermore, endothelial injury facilitates LDL oxidation and subendothelial deposition, amplifying inflammatory responses. Macrophages uptake ox-LDL, transforming into foam cells that accumulate within the tunica intima and contribute to the formation of fatty streaks. Macrophages exhibit functional plasticity, polarizing into pro-inflammatory M1 or anti-inflammatory M2 phenotypes in response to the changing microenvironment. This course critically influences the inflammatory status and plaque stability. Under pathological conditions, VSMCs change from a contractile to a synthetic state, participating in plaque remodeling and fibrous cap formation. Additionally, VSMCs can internalize ox-LDL to form foam cells, further exacerbating plaque progression.

Dysregulation of glucose and lipid metabolism profoundly impacts the function of ECs, macrophages, and VSMCs in atherosclerosis. ECs depend on glycolysis and fatty acid oxidation to support angiogenesis, migration, and barrier function. Metabolic reprogramming in these cells alters the plaque microenvironment and influences disease progression. In macrophages, foam cell formation is directly driven by disrupted cholesterol uptake and efflux, alongside inflammatory activation fueled by glycolytic shifts. Similarly, VSMC phenotypic modulation is closely linked to metabolic alterations, including increased glycolysis and impaired mitochondrial respiration. Therefore, it is crucial to thoroughly investigate the process of lipid and glucose metabolism in these cells to create novel therapeutic approaches.

Emerging technologies, such as single-cell RNA sequencing and spatial transcriptomics, have enabled deeper investigation into the cellular heterogeneity and molecular interactions within atherosclerotic plaques. These advances provide new insights into disease mechanisms and facilitate the identification of novel therapeutic targets. Future efforts should prioritize multi-target strategies and personalized treatment approaches. Further research is also needed to elucidate the effects of existing cardioprotective drugs, such as SGLT2 inhibitors and GLP-1 receptor agonists, on cellular metabolism within plaques.

In conclusion, understanding the metabolic reprogramming of vascular and immune cells provides a renewed perspective on atherosclerosis pathogenesis. Integrating multi-omics data with mechanistic studies will be essential for developing innovative therapies that restore metabolic homeostasis and promote plaque stability.

## Figures and Tables

**Table 1:** Traditional Clinical Medicine for Atherosclerosis.

Traditional Clinical Medicine	
Lipid control [113]	Statins and Fibrates
	Ezetimibe
	PCSK9 inhibitor
Antiplatelet therapy	Aspirin
	P2Y12 receptor inhibitors: clopidogrel
Anti-inflammatory treatment [114]	Reduce monocyte recruitment
	Inhibit neutrophil extracellular traps (NETs)
	Suppress immune checkpoints
	Stimulate inflammation to subside
Antidiabetic treatment [115]	Insulin
	Biguanides
	Thiazolidinediones(TZDs)
	Glucagon-like peptide 1 (GLP-1) receptor agonist
	Sodium-glucose cotransporter protein-2 (SGLT-2) inhibitors

**Table 2:** Potential Possible Therapeutic Targets.

Potential Possible Therapeutic Targets [116]		
Therapy targeting glycolysis	miR-638(Target: LDHA)	Inhibit VSMC proliferation and migration
	2-deoxyglucose /2DG(Target: glycolysis in macrophage)	Decrease the production of the inflammatory factor IL-1β
	Lactate transport proteins	Influence lactate production
Therapy targeting lipid metabolism	Omega-3 fatty acids	Decrease TRL
	Antisense oligonucleotides (Target: APO3)	
	Cholesteryl ester transfer protein inhibitors	Increase HDL-C
	Recombinant HDL infusion	
Others [117]	Targeted pro-inflammatory cascade therapy for inflammation; clonal hematopoietic; intestinal microbiota therapy, etc.	

## Data Availability

Not applicable.

## References

[1] Summerhill V.I., Grechko A.V., Yet S.-F., Sobenin I.A., Orekhov A.N. (2019). The Atherogenic Role of Circulating Modified Lipids in Atherosclerosis. Int. J. Mol. Sci..

[2] Libby P. (2021). The Changing Landscape of Atherosclerosis. Nature.

[3] Soehnlein O., Libby P. (2021). Targeting Inflammation in Atherosclerosis—From Experimental Insights to the Clinic. Nat. Rev. Drug Discov..

[4] Vilela E., Bettencourt N. (2025). From Cholesterol Metabolism to Comprehensive Lipid Management and Crosstalk of Inflammation: Expanding the Frontiers of Cardiovascular Prevention. Arq. Bras. Cardiol..

[5] Liu A., Zhang Y., Xun S., Sun M. (2022). Trimethylamine N-Oxide Promotes Atherosclerosis via Regulating the Enriched Abundant Transcript 1/miR-370-3p/Signal Transducer and Activator of Transcription 3/Flavin-Containing Monooxygenase-3 Axis. Bioengineered.

[6] Austin R.C., Lentz S.R., Werstuck G.H. (2004). Role of Hyperhomocysteinemia in Endothelial Dysfunction and Atherothrombotic Disease. Cell Death Differ..

[7] Chen W., Li Z., Zhao Y., Chen Y., Huang R. (2023). Global and National Burden of Atherosclerosis from 1990 to 2019: Trend Analysis Based on the Global Burden of Disease Study 2019. Chin. Med. J..

[8] Xu R., Yuan W., Wang Z. (2023). Advances in Glycolysis Metabolism of Atherosclerosis. J. Cardiovasc. Trans. Res..

[9] Gimbrone M.A., García-Cardeña G. (2016). Endothelial Cell Dysfunction and the Pathobiology of Atherosclerosis. Circ. Res..

[10] Li H., Förstermann U., Xia N., Kuntic M., Münzel T., Daiber A. (2025). Pharmacological Targeting of Endothelial Nitric Oxide Synthase Dysfunction and Nitric Oxide Replacement Therapy. Free. Radic. Biol. Med..

[11] Zhang X., Chen B., Wu J., Sha J., Yang B., Zhu J., Sun J., Hartung J., Bao E. (2020). Aspirin Enhances the Protection of Hsp90 from Heat-Stressed Injury in Cardiac Microvascular Endothelial Cells Through PI3K-Akt and PKM2 Pathways. Cells.

[12] Zhang R., Li R., Liu Y., Li L., Tang Y. (2019). The Glycolytic Enzyme PFKFB3 Controls TNF-α-Induced Endothelial Proinflammatory Responses. Inflammation.

[13] Zhili G., Zhimin G., Nenghua Z. (2025). Role of Vascular Endothelial Dysfunction and Metabolic Eprogramming of Immune Cells in the Development and Ogression of Atherosclerosis. Front. Physiol..

[14] Tamargo I.A., Baek K.I., Kim Y., Park C., Jo H. (2023). Flow-Induced Reprogramming of Endothelial Cells in Atherosclerosis. Nat. Rev. Cardiol..

[15] Sakurai Y., Yu L., Matsuda A., Maishi N., Hida K. (2025). Vascular Inflammation and Cancer Malignancy. J. Oral Biosci..

[16] De Bock K., Georgiadou M., Schoors S., Kuchnio A., Wong B.W., Cantelmo A.R., Quaegebeur A., Ghesquière B., Cauwenberghs S., Eelen G. (2013). Role of PFKFB3-Driven Glycolysis in Vessel Sprouting. Cell.

[17] Eelen G., de Zeeuw P., Simons M., Carmeliet P. (2015). Endothelial Cell Metabolism in Normal and Diseased Vasculature. Circ. Res..

[18] Zhou L., Li J., Wang J., Niu X., Li J., Zhang K. (2024). Pathogenic Role of PFKFB3 in Endothelial Inflammatory Diseases. Front. Mol. Biosci..

[19] Kuo A., Lee M.Y., Sessa W.C. (2017). Lipid Droplet Biogenesis and Function in the Endothelium. Circ. Res..

[20] Kalluri A.S., Vellarikkal S.K., Edelman E.R., Nguyen L., Subramanian A., Ellinor P.T., Regev A., Kathiresan S., Gupta R.M. (2019). Single Cell Analysis of the Normal Mouse Aorta Reveals Functionally Distinct Endothelial Cell Populations. Circulation.

[21] Kim B., Arany Z. (2022). Endothelial Lipid Metabolism. Cold Spring Harb. Perspect. Med..

[22] Boutagy N.E., Gamez-Mendez A., Fowler J.W., Zhang H., Chaube B.K., Esplugues E., Kuo A., Lee S., Horikami D., Zhang J. (2024). Dynamic Metabolism of Endothelial Triglycerides Protects against Atherosclerosis in Mice. J. Clin. Investig..

[23] Inoue T., Kobayashi K., Inoguchi T., Sonoda N., Fujii M., Maeda Y., Fujimura Y., Miura D., Hirano K., Takayanagi R. (2011). Reduced Expression of Adipose Triglyceride Lipase Enhances Tumor Necrosis Factor α-Induced Intercellular Adhesion Molecule-1 Expression in Human Aortic Endothelial Cells via Protein Kinase C-Dependent Activation of Nuclear Factor-κB. J. Biol. Chem..

[24] Abumrad N.A., Cabodevilla A.G., Samvoski D., Pietka T., Basu D., Goldberg I.J. (2021). Endothelial Cell Receptors in Tissue Lipid Uptake and Metabolism. Circ. Res..

[25] Izquierdo M.C., Cabodevilla A.G., Basu D., Nasias D., Kanter J.E., Ho W., Gjini J., Fisher E.A., Kim J., Lee W. (2024). Hyperchylomicronemia Causes Endothelial Cell Inflammation and Increases Atherosclerosis. Res. Sq..

[26] Singh B., Kosuru R., Lakshmikanthan S., Sorci-Thomas M., Zhang D., Sparapani R., Vasquez-Vivar J., Chrzanowska M. (2021). Endothelial Rap1 Restricts Inflammatory Signaling to Protect from the Progression of Atherosclerosis. Arterioscler. Thromb. Vasc. Biol..

[27] Sarrazy V., Viaud M., Westerterp M., Ivanov S., Giorgetti-Peraldi S., Guinamard R., Gautier E.L., Thorp E.B., De Vivo D.C., Yvan-Charvet L. (2016). Disruption of Glut1 in Hematopoietic Stem Cells Prevents Myelopoiesis and Enhanced Glucose Flux in Atheromatous Plaques of ApoE(-/-) Mice. Circ. Res..

[28] Kim J., Gao P., Liu Y.-C., Semenza G.L., Dang C.V. (2007). Hypoxia-Inducible Factor 1 and Dysregulated c-Myc Cooperatively Induce Vascular Endothelial Growth Factor and Metabolic Switches Hexokinase 2 and Pyruvate Dehydrogenase Kinase 1. Mol. Cell. Biol..

[29] Song J., Li Y., Song J., Hou F., Liu B., Li A. (2017). Mangiferin Protects Mitochondrial Function by Preserving Mitochondrial Hexokinase-II in Vessel Endothelial Cells. Biochim. Biophys. Acta (BBA)—Mol. Basis Dis..

[30] Doddaballapur A., Michalik K.M., Manavski Y., Lucas T., Houtkooper R.H., You X., Chen W., Zeiher A.M., Potente M., Dimmeler S. (2015). Laminar Shear Stress Inhibits Endothelial Cell Metabolism via KLF2-Mediated Repression of PFKFB3. Arterioscler. Thromb. Vasc. Biol..

[31] Wik J.A., Lundbäck P., Poulsen L.l.C., Haraldsen G., Skålhegg B.S., Hol J. (2020). 3PO Inhibits Inflammatory NFκB and Stress-Activated Kinase Signaling in Primary Human Endothelial Cells Independently of Its Target PFKFB3. PLoS ONE.

[32] Yang Q., Xu J., Ma Q., Liu Z., Sudhahar V., Cao Y., Wang L., Zeng X., Zhou Y., Zhang M. (2018). PRKAA1/AMPKα1-Driven Glycolysis in Endothelial Cells Exposed to Disturbed Flow Protects against Atherosclerosis. Nat. Commun..

[33] Lü S., Deng J., Liu H., Liu B., Yang J., Miao Y., Li J., Wang N., Jiang C., Xu Q. (2018). PKM2-Dependent Metabolic Reprogramming in CD4+ T Cells Is Crucial for Hyperhomocysteinemia-Accelerated Atherosclerosis. J. Mol. Med..

[34] Xu R.-H., Liu B., Wu J.-D., Yan Y.-Y., Wang J.-N. (2016). miR-143 Is Involved in Endothelial Cell Dysfunction through Suppression of Glycolysis and Correlated with Atherosclerotic Plaques Formation. Eur. Rev. Med. Pharmacol. Sci..

[35] Zhao X., Tan F., Cao X., Cao Z., Li B., Shen Z., Tian Y. (2019). PKM2-Dependent Glycolysis Promotes the Proliferation and Migration of Vascular Smooth Muscle Cells during Atherosclerosis. Acta Biochim. Biophys. Sin..

[36] Slawson C., Copeland R.J., Hart G.W. (2010). O-GlcNAc Signaling: A Metabolic Link between Diabetes and Cancer?. Trends Biochem. Sci..

[37] Vizán P., Sánchez-Tena S., Alcarraz-Vizán G., Soler M., Messeguer R., Pujol M.D., Lee W.-N.P., Cascante M. (2009). Characterization of the Metabolic Changes Underlying Growth Factor Angiogenic Activation: Identification of New Potential Therapeutic Targets. Carcinogenesis.

[38] Shantha G.P.S., Wasserman B., Astor B.C., Coresh J., Brancati F., Sharrett A.R., Young J.H. (2013). Association of Blood Lactate with Carotid Atherosclerosis: The Atherosclerosis Risk in Communities (ARIC) Carotid MRI Study. Atherosclerosis.

[39] Manosalva C., Quiroga J., Hidalgo A.I., Alarcón P., Anseoleaga N., Hidalgo M.A., Burgos R.A. (2022). Role of Lactate in Inflammatory Processes: Friend or Foe. Front. Immunol..

[40] Brown T.P., Ganapathy V. (2020). Lactate/GPR81 Signaling and Proton Motive Force in Cancer: Role in Angiogenesis, Immune Escape, Nutrition, and Warburg Phenomenon. Pharmacol. Ther..

[41] Van Den Oord S.C.H., Akkus Z., Renaud G., Bosch J.G., Van Der Steen A.F.W., Sijbrands E.J.G., Schinkel A.F.L. (2014). Assessment of Carotid Atherosclerosis, Intraplaque Neovascularization, and Plaque Ulceration Using Quantitative Contrast-Enhanced Ultrasound in Asymptomatic Patients with Diabetes Mellitus. Eur. Heart J. Cardiovasc. Imaging.

[42] Sun Z., Han Y., Song S., Chen T., Han Y., Liu Y. (2019). Activation of GPR81 by Lactate Inhibits Oscillatory Shear Stress-induced Endothelial Inflammation by Activating the Expression of KLF2. IUBMB Life.

[43] Ramji D.P., Davies T.S. (2015). Cytokines in Atherosclerosis: Key Players in All Stages of Disease and Promising Therapeutic Targets. Cytokine Growth Factor Rev..

[44] Tedgui A., Mallat Z. (2006). Cytokines in Atherosclerosis: Pathogenic and Regulatory Pathways. Physiol. Rev..

[45] Wang R., Wang M., Ye J., Sun G., Sun X. (2020). Mechanism Overview and Target Mining of Atherosclerosis: Endothelial Cell Injury in Atherosclerosis Is Regulated by Glycolysis (Review). Int. J. Mol. Med..

[46] Jinnouchi H., Guo L., Sakamoto A., Torii S., Sato Y., Cornelissen A., Kuntz S., Paek K.H., Fernandez R., Fuller D. (2019). Diversity of Macrophage Phenotypes and Responses in Atherosclerosis. Cell Mol. Life Sci..

[47] O’Neill L.A.J., Pearce E.J. (2016). Immunometabolism Governs Dendritic Cell and Macrophage Function. J. Exp. Med..

[48] Yurdagul A., Finney A.C., Woolard M.D., Orr A.W. (2016). The Arterial Microenvironment: The Where and Why of Atherosclerosis. Biochem. J..

[49] Wu J., He S., Song Z., Chen S., Lin X., Sun H., Zhou P., Peng Q., Du S., Zheng S. (2023). Macrophage Polarization States in Atherosclerosis. Front. Immunol..

[50] Eshghjoo S., Kim D.M., Jayaraman A., Sun Y., Alaniz R.C. (2022). Macrophage Polarization in Atherosclerosis. Genes.

[51] Yu L., Zhang Y., Liu C., Wu X., Wang S., Sui W., Zhang Y., Zhang C., Zhang M. (2023). Heterogeneity of Macrophages in Atherosclerosis Revealed by Single-cell RNA Sequencing. FASEB J..

[52] Huang S.C.-C., Everts B., Ivanova Y., O’Sullivan D., Nascimento M., Smith A.M., Beatty W., Love-Gregory L., Lam W.Y., O’Neill C.M. (2014). Cell-Intrinsic Lysosomal Lipolysis Is Essential for Alternative Activation of Macrophages. Nat. Immunol..

[53] Wei X., Song H., Yin L., Rizzo M.G., Sidhu R., Covey D.F., Ory D.S., Semenkovich C.F. (2016). Fatty Acid Synthesis Configures the Plasma Membrane for Inflammation in Diabetes. Nature.

[54] Olefsky J.M., Glass C.K. (2010). Macrophages, Inflammation, and Insulin Resistance. Annu. Rev. Physiol..

[55] O’Neill L.A.J., Kishton R.J., Rathmell J. (2016). A Guide to Immunometabolism for Immunologists. Nat. Rev. Immunol..

[56] Mitra S., Goyal T., Mehta J.L. (2011). Oxidized LDL, LOX-1 and Atherosclerosis. Cardiovasc. Drugs Ther..

[57] Pirillo A., Norata G.D., Catapano A.L. (2013). LOX-1, OxLDL, and Atherosclerosis. Mediat. Inflamm..

[58] Von Eckardstein A., Kardassis D. (2015). High Density Lipoproteins: From Biological Understanding to Clinical Exploitation.

[59] Doddapattar P., Dev R., Ghatge M., Patel R.B., Jain M., Dhanesha N., Lentz S.R., Chauhan A.K. (2022). Myeloid Cell PKM2 Deletion Enhances Efferocytosis and Reduces Atherosclerosis. Circ. Res..

[60] Li Q., Leng K., Liu Y., Sun H., Gao J., Ren Q., Zhou T., Dong J., Xia J. (2020). The Impact of Hyperglycaemia on PKM2-Mediated NLRP3 Inflammasome/Stress Granule Signalling in Macrophages and Its Correlation with Plaque Vulnerability: An in Vivo and in Vitro Study. Metabolism.

[61] Shirai T., Nazarewicz R.R., Wallis B.B., Yanes R.E., Watanabe R., Hilhorst M., Tian L., Harrison D.G., Giacomini J.C., Assimes T.L. (2016). The Glycolytic Enzyme PKM2 Bridges Metabolic and Inflammatory Dysfunction in Coronary Artery Disease. J. Exp. Med..

[62] Rayner K.J., Suárez Y., Dávalos A., Parathath S., Fitzgerald M.L., Tamehiro N., Fisher E.A., Moore K.J., Fernández-Hernando C. (2010). MiR-33 Contributes to the Regulation of Cholesterol Homeostasis. Science.

[63] Vickers K.C., Shoucri B.M., Levin M.G., Wu H., Pearson D.S., Osei-Hwedieh D., Collins F.S., Remaley A.T., Sethupathy P. (2013). MicroRNA-27b Is a Regulatory Hub in Lipid Metabolism and Is Altered in Dyslipidemia. Hepatology.

[64] Koelwyn G.J., Corr E.M., Erbay E., Moore K.J. (2018). Regulation of Macrophage Immunometabolism in Atherosclerosis. Nat. Immunol..

[65] Jha A.K., Huang S.C.-C., Sergushichev A., Lampropoulou V., Ivanova Y., Loginicheva E., Chmielewski K., Stewart K.M., Ashall J., Everts B. (2015). Network Integration of Parallel Metabolic and Transcriptional Data Reveals Metabolic Modules That Regulate Macrophage Polarization. Immunity.

[66] Tannahill G.M., Curtis A.M., Adamik J., Palsson-McDermott E.M., McGettrick A.F., Goel G., Frezza C., Bernard N.J., Kelly B., Foley N.H. (2013). Succinate Is an Inflammatory Signal That Induces IL-1β through HIF-1α. Nature.

[67] Clementi E., Brown G.C., Feelisch M., Moncada S. (1998). Persistent Inhibition of Cell Respiration by Nitric Oxide: Crucial Role of *S*-Nitrosylation of Mitochondrial Complex I and Protective Action of Glutathione. Proc. Natl. Acad. Sci. USA.

[68] Rodríguez-Prados J.-C., Través P.G., Cuenca J., Rico D., Aragonés J., Martín-Sanz P., Cascante M., Boscá L. (2010). Substrate Fate in Activated Macrophages: A Comparison between Innate, Classic, and Alternative Activation. J. Immunol..

[69] Rius J., Guma M., Schachtrup C., Akassoglou K., Zinkernagel A.S., Nizet V., Johnson R.S., Haddad G.G., Karin M. (2008). NF-κB Links Innate Immunity to the Hypoxic Response through Transcriptional Regulation of HIF-1α. Nature.

[70] Xiao Q., Hou R., Xie L., Niu M., Pan X., Zhu X. (2023). Macrophage Metabolic Reprogramming and Atherosclerotic Plaque Microenvironment: Fostering Each Other?. Clin. Transl. Med..

[71] Palsson-McDermott E.M., Curtis A.M., Goel G., Lauterbach M.A., Sheedy F.J., Gleeson L.E., van den Bosch M.W., Quinn S.R., Domingo-Fernandez R., Johnson D.G. (2015). Pyruvate Kinase M2 Regulates Hif-1α Activity and IL-1β Induction, and Is a Critical Determinant of the Warburg Effect in LPS-Activated Macrophages. Cell Metab..

[72] Colegio O.R., Chu N.-Q., Szabo A.L., Chu T., Rhebergen A.M., Jairam V., Cyrus N., Brokowski C.E., Eisenbarth S.C., Phillips G.M. (2014). Functional Polarization of Tumour-Associated Macrophages by Tumour-Derived Lactic Acid. Nature.

[73] Liu N., Luo J., Kuang D., Xu S., Duan Y., Xia Y., Wei Z., Xie X., Yin B., Chen F. (2019). Lactate Inhibits ATP6V0d2 Expression in Tumor-Associated Macrophages to Promote HIF-2**α**–Mediated Tumor Progression. J. Clin. Investig..

[74] Khazaei M., Nematbakhsh M. (2012). Experimental Research Effect of Experimentally Induced Metabolic Acidosis on Aortic Endothelial Permeability and Serum Nitric Oxide Concentration in Normal and High-Cholesterol Fed Rabbits. Arch. Med. Sci..

[75] Hoque R., Farooq A., Ghani A., Gorelick F., Mehal W.Z. (2014). Lactate Reduces Liver and Pancreatic Injury in Toll-Like Receptor– and Inflammasome-Mediated Inflammation via GPR81-Mediated Suppression of Innate Immunity. Gastroenterology.

[76] Yang K., Xu J., Fan M., Tu F., Wang X., Ha T., Williams D.L., Li C. (2020). Lactate Suppresses Macrophage Pro-Inflammatory Response to LPS Stimulation by Inhibition of YAP and NF-κB Activation via GPR81-Mediated Signaling. Front. Immunol..

[77] Bohn T., Rapp S., Luther N., Klein M., Bruehl T.-J., Kojima N., Aranda Lopez P., Hahlbrock J., Muth S., Endo S. (2018). Tumor Immunoevasion via Acidosis-Dependent Induction of Regulatory Tumor-Associated Macrophages. Nat. Immunol..

[78] Hanssen N.M.J., Wouters K., Huijberts M.S., Gijbels M.J., Sluimer J.C., Scheijen J.L.J.M., Heeneman S., Biessen E.A.L., Daemen M.J.A.P., Brownlee M. (2014). Higher Levels of Advanced Glycation Endproducts in Human Carotid Atherosclerotic Plaques Are Associated with a Rupture-Prone Phenotype. Eur. Heart J..

[79] Park H.Y., Kim M.-J., Lee S., Jin J., Lee S., Kim J.-G., Choi Y.-K., Park K.-G. (2021). Inhibitory Effect of a Glutamine Antagonist on Proliferation and Migration of VSMCs via Simultaneous Attenuation of Glycolysis and Oxidative Phosphorylation. Int. J. Mol. Sci..

[80] Shi J., Yang Y., Cheng A., Xu G., He F. (2020). Metabolism of Vascular Smooth Muscle Cells in Vascular Diseases. Am. J. Physiol. Heart Circ. Physiol..

[81] Grootaert M.O.J., Bennett M.R. (2021). Vascular Smooth Muscle Cells in Atherosclerosis: Time for a Re-Assessment. Cardiovasc. Res..

[82] Grootaert M.O.J., Moulis M., Roth L., Martinet W., Vindis C., Bennett M.R., De Meyer G.R.Y. (2018). Vascular Smooth Muscle Cell Death, Autophagy and Senescence in Atherosclerosis. Cardiovasc. Res..

[83] Campbell J.H., Campbell G.R. (2012). Smooth Muscle Phenotypic Modulation—A Personal Experience. Arter. Thromb. Vasc. Biol..

[84] Regan C.P., Adam P.J., Madsen C.S., Owens G.K. (2000). Molecular Mechanisms of Decreased Smooth Muscle Differentiation Marker Expression after Vascular Injury. J. Clin. Investig..

[85] Li S., Wang D.-Z., Wang Z., Richardson J.A., Olson E.N. (2003). The Serum Response Factor Coactivator Myocardin Is Required for Vascular Smooth Muscle Development. Proc. Natl. Acad. Sci. USA.

[86] Johnson J.L. (2017). Metalloproteinases in Atherosclerosis. Eur. J. Pharmacol..

[87] Langley S.R., Willeit K., Didangelos A., Matic L.P., Skroblin P., Barallobre-Barreiro J., Lengquist M., Rungger G., Kapustin A., Kedenko L. (2017). Extracellular Matrix Proteomics Identifies Molecular Signature of Symptomatic Carotid Plaques. J. Clin. Investig..

[88] Allahverdian S., Chaabane C., Boukais K., Francis G.A., Bochaton-Piallat M.-L. (2018). Smooth Muscle Cell Fate and Plasticity in Atherosclerosis. Cardiovasc. Res..

[89] Orr A.W., Hastings N.E., Blackman B.R., Wamhoff B.R. (2009). Complex Regulation and Function of the Inflammatory Smooth Muscle Cell Phenotype in Atherosclerosis. J. Vasc. Res..

[90] Kapustin A.N., Chatrou M.L.L., Drozdov I., Zheng Y., Davidson S.M., Soong D., Furmanik M., Sanchis P., De Rosales R.T.M., Alvarez-Hernandez D. (2015). Vascular Smooth Muscle Cell Calcification Is Mediated by Regulated Exosome Secretion. Circ. Res..

[91] Zuccolo E., Badi I., Scavello F., Gambuzza I., Mancinelli L., Macrì F., Tedesco C.C., Veglia F., Bonfigli A.R., Olivieri F. (2020). The microRNA-34a-Induced Senescence-Associated Secretory Phenotype (SASP) Favors Vascular Smooth Muscle Cells Calcification. Int. J. Mol. Sci..

[92] Kim J.-H., Bae K.-H., Byun J.-K., Lee S., Kim J.-G., Lee I.K., Jung G.-S., Lee Y.M., Park K.-G. (2017). Lactate Dehydrogenase-A Is Indispensable for Vascular Smooth Muscle Cell Proliferation and Migration. Biochem. Biophys. Res. Commun..

[93] Zhu Y., Han X.-Q., Sun X.-J., Yang R., Ma W.-Q., Liu N.-F. (2020). Lactate Accelerates Vascular Calcification through NR4A1-Regulated Mitochondrial Fission and BNIP3-Related Mitophagy. Apoptosis.

[94] Umetani M., Ghosh P., Ishikawa T., Umetani J., Ahmed M., Mineo C., Shaul P.W. (2014). The Cholesterol Metabolite 27-Hydroxycholesterol Promotes Atherosclerosis via Proinflammatory Processes Mediated by Estrogen Receptor Alpha. Cell Metab..

[95] Yu L., Xu L., Chu H., Peng J., Sacharidou A., Hsieh H., Weinstock A., Khan S., Ma L., Durán J.G.B. (2023). Macrophage-to-Endothelial Cell Crosstalk by the Cholesterol Metabolite 27HC Promotes Atherosclerosis in Male Mice. Nat. Commun..

[96] Park D., Han C., Elliott M.R., Kinchen J.M., Trampont P.C., Das S., Collins S., Lysiak J.J., Hoehn K.L., Ravichandran K.S. (2011). Continued Clearance of Apoptotic Cells Critically Depends on the Phagocyte Ucp2 Protein. Nature.

[97] Ruan G.-X., Kazlauskas A. (2013). Lactate Engages Receptor Tyrosine Kinases Axl, Tie2, and Vascular Endothelial Growth Factor Receptor 2 to Activate Phosphoinositide 3-Kinase/Akt and Promote Angiogenesis. J. Biol. Chem..

[98] Zhu Y., Ji J.-J., Yang R., Han X.-Q., Sun X.-J., Ma W.-Q., Liu N.-F. (2019). Lactate Accelerates Calcification in VSMCs through Suppression of BNIP3-Mediated Mitophagy. Cell. Signal..

[99] Stachteas P., Karakasis P., Patoulias D., Clemenza F., Fragakis N., Rizzo M. (2024). The Effect of Sodium-Glucose Co-Transporter-2 Inhibitors on Markers of Subclinical Atherosclerosis. Ann. Med..

[100] Libby P., Buring J.E., Badimon L., Hansson G.K., Deanfield J., Bittencourt M.S., Tokgözoğlu L., Lewis E.F. (2019). Atherosclerosis. Nat. Rev. Dis. Primers.

[101] Collins R., Reith C., Emberson J., Armitage J., Baigent C., Blackwell L., Blumenthal R., Danesh J., Smith G.D., DeMets D. (2016). Interpretation of the Evidence for the Efficacy and Safety of Statin Therapy. Lancet.

[102] Hammersley D., Signy M. (2016). Ezetimibe: An Update on Its Clinical Usefulness in Specific Patient Groups. Ther. Adv. Chronic Dis..

[103] Barale C., Melchionda E., Morotti A., Russo I. (2021). PCSK9 Biology and Its Role in Atherothrombosis. Int. J. Mol. Sci..

[104] Soehnlein O., Drechsler M., Döring Y., Lievens D., Hartwig H., Kemmerich K., Ortega-Gómez A., Mandl M., Vijayan S., Projahn D. (2013). Distinct Functions of Chemokine Receptor Axes in the Atherogenic Mobilization and Recruitment of Classical Monocytes. EMBO Mol. Med..

[105] Chiang N., Fredman G., Bäckhed F., Oh S.F., Vickery T., Schmidt B.A., Serhan C.N. (2012). Infection Regulates Pro-Resolving Mediators That Lower Antibiotic Requirements. Nature.

[106] Adler A.I., Stratton I.M., Neil H.A.W., Yudkin J.S., Matthews D.R., Cull C.A., Wright A.D., Turner R.C., Holman R.R. (2000). Association of Systolic Blood Pressure with Macrovascular and Microvascular Complications of Type 2 Diabetes (UKPDS 36): Prospective Observational Study. BMJ.

[107] Stroope C., Nettersheim F.S., Coon B., Finney A.C., Schwartz M.A., Ley K., Rom O., Arif Yurdagul J. (2024). Dysregulated Cellular Metabolism in Atherosclerosis: Mediators and Therapeutic Opportunities. Nat. Metab..

[108] Nissen S.E., Nicholls S.J., Wolski K., Nesto R., Kupfer S., Perez A., Jure H., De Larochellière R., Staniloae C.S., Mavromatis K. (2008). Comparison of Pioglitazone vs Glimepiride on Progression of Coronary Atherosclerosis in Patients with Type 2 Diabetes: The PERISCOPE Randomized Controlled Trial. JAMA.

[109] Liu Y., Chen X., Li J. (2017). Resveratrol Protects against Oxidized Low-density Lipoprotein-induced Human Umbilical Vein Endothelial Cell Apoptosis via Inhibition of Mitochondrial-derived Oxidative Stress. Mol. Med. Rep..

[110] Choi H.M., Doss H.M., Kim K.S. (2020). Multifaceted Physiological Roles of Adiponectin in Inflammation and Diseases. Int. J. Mol. Sci..

[111] Sattar N., Lee M.M.Y., Kristensen S.L., Branch K.R.H., Del Prato S., Khurmi N.S., Lam C.S.P., Lopes R.D., McMurray J.J.V., Pratley R.E. (2021). Cardiovascular, Mortality, and Kidney Outcomes with GLP-1 Receptor Agonists in Patients with Type 2 Diabetes: A Systematic Review and Meta-Analysis of Randomised Trials. Lancet Diabetes Endocrinol..

[112] Jojima T., Uchida K., Akimoto K., Tomotsune T., Yanagi K., Iijima T., Suzuki K., Kasai K., Aso Y. (2017). Liraglutide, a GLP-1 Receptor Agonist, Inhibits Vascular Smooth Muscle Cell Proliferation by Enhancing AMP-Activated Protein Kinase and Cell Cycle Regulation, and Delays Atherosclerosis in *ApoE* Deficient Mice. Atherosclerosis.

[113] Jianu N., Nițu E.-T., Merlan C., Nour A., Buda S., Suciu M., Luca S.A., Sbârcea L., Andor M., Buda V. (2025). A Comprehensive Review of the Latest Approaches to Managing Hypercholesterolemia: A Comparative Analysis of Conventional and Novel Treatments: Part II. Pharmaceuticals.

[114] Hudson A., Igiehon O.O., Woolard M.D., Yurdagul A. (2025). Anti-Atherogenic Mechanisms and Therapies. Curr. Atheroscler. Rep..

[115] Ma X., Liu Z., Ilyas I., Little P.J., Kamato D., Sahebka A., Chen Z., Luo S., Zheng X., Weng J. (2021). GLP-1 Receptor Agonists (GLP-1RAs): Cardiovascular Actions and Therapeutic Potential. Int. J. Biol. Sci..

[116] Tokgözoğlu L., Libby P. (2022). The Dawn of a New Era of Targeted Lipid-Lowering Therapies. Eur. Heart J..

[117] Ait-Oufella H., Libby P. (2024). Inflammation and Atherosclerosis: Prospects for Clinical Trials. Arterioscler. Thromb. Vasc. Biol..

[118] Ali L., Schnitzler J.G., Kroon J. (2018). Metabolism: The Road to Inflammation and Atherosclerosis. Curr. Opin. Lipidol..

[119] Chen S., Chen H., Yu C., Lu R., Song T., Wang X., Tang W., Gao Y. (2019). MiR-638 Repressed Vascular Smooth Muscle Cell Glycolysis by Targeting LDHA. Open Med..

[120] Zheng W.C., Chan W., Dart A., Shaw J.A. (2024). Novel Therapeutic Targets and Emerging Treatments for Atherosclerotic Cardiovascular Disease. Eur. Heart J. Cardiovasc. Pharmacother..

